# Lipid Lowering Drugs: Present Status and Future Developments

**DOI:** 10.1007/s11883-021-00918-3

**Published:** 2021-03-10

**Authors:** Massimiliano Ruscica, Nicola Ferri, Raul D. Santos, Cesare R. Sirtori, Alberto Corsini

**Affiliations:** 1 Department of Pharmacological and Biomolecular Sciences, Università degli Studi di Milano, Milan, Italy; 2 Department of Pharmaceutical and Pharmacological Sciences, Università degli Studi di Padova, Padova, Italy; 3 Lipid Clinic, Heart Institute (InCor), University of Sao Paulo, São Paulo, Brazil; 4 Hospital Israelita Albert Einstein, São Paulo, Brazil; 5 IRCCS MultiMedica, Sesto S. Giovanni, Milan, Italy

**Keywords:** APOCIII-L_Rx_, ANGPTL3L_RX_, Inclisiran, Lipoprotein(a), Proprotein convertase subtilisin/kexin type 9

## Abstract

**Purpose of review:**

Based on the recent data of the DA VINCI study, it is clear that, besides utilization of statins, there is a need to increase non-statin lipid lowering approaches to reduce the cardiovascular burden in patients at highest risk.

**Recent findings:**

For hypercholesterolemia, the small synthetic molecule bempedoic acid has the added benefit of selective liver activation, whereas inclisiran, a hepatic inhibitor of the PCSK9 synthesis, has comparable effects with PCSK9 monoclonal antibodies. For hypertriglyceridemia, cardiovascular benefit has been achieved by the use of icosapent ethyl, whereas results with pemafibrate, a selective agonist of PPAR-α, are eagerly awaited. In the era of RNA-based therapies, new options are offered to dramatically reduce levels of lipoprotein(a) (APO(a)L_RX_) and of triglycerides (ANGPTL3L_RX_ and APOCIII-L_Rx_).

**Summary:**

Despite the demonstrated benefits of statins, a large number of patients still remain at significant risk because of inadequate LDL-C reduction or elevated blood triglyceride-rich lipoproteins or lipoprotein(a). The area of lipid modulating agents is still ripe with ideas and major novelties are to be awaited in the next few years.

## Introduction

Atherosclerotic cardiovascular disease (ASCVD) encompassing pathologies caused by atherosclerosis within the coronary, cerebral, and peripheral arteries and the aorta is a leading cause of death and disability worldwide [1]. Among the risk factors accounting for this condition (nine have been identified in the INTERHEART study), lipid/lipoprotein abnormalities play a central role in this process [2]. Although epidemiologic, genetic, and clinical intervention studies have unquestionably identified low-density lipoprotein cholesterol (LDL-C) to be causal in this process [3, 4], the contribution of triglycerides (TG) to cardiovascular risk is also evident from long-term prospective and genetic studies [5, 6].

While the scientific evidence for reducing LDL-C is definitely strong, i.e., the more LDL-C is reduced the larger will be the ASCVD risk reduction [7, 8], a substantial residual risk persists. This is especially evident among high risk subjects with familial combined hyperlipidemia, familial hypertriglyceridemia as well as in those with the metabolic syndrome, type 2 diabetes mellitus (T2DM) and the related atherogenic dyslipidemia (reviewed in [9, 10]). This last is characterized by elevated serum TG, increased remnant lipoproteins, and low levels of high-density lipoprotein cholesterol (HDL-C) [11]. Very recently, hypertriglyceridemia in statin-treated T2DM patients was directly associated with a raised ASCVD risk after coronary revascularization [12]*.* Although not in the remit of this review article, it is worth mentioning that the elevated risk conferred by reduced HDL-C levels has not been as yet fully addressed by targeted therapies, whereas administration of recombinant HDL in different forms is still undergoing clinical evaluation [13, 14].

Finally, more extensive epidemiological evaluation of the lipoprotein profile of high-risk patients also brought attention to elevated levels of lipoprotein(a) [Lp(a)], a lipoprotein composed of an LDL-like particle, covalently bound to apo(a) and associated with a raised ASCVD risk [15]. On an equimolar basis, Lp(a) is more atherogenic than LDL because it carries all the proatherogenic components of LDL as well as apo(a), that binds phosphocholine containing oxidized phospholipids [16]. Apo(a) seems to mediate atherothrombosis by enhancing inflammation and by potential antifibrinolytic effects (e.g., inhibition of plasminogen activation and of fibrin degradation) [17].

Thus, the present review will focus on the current status of novel pharmacological approaches to these more recently investigated lipoprotein abnormalities. Some of the described newer drugs affect more than one lipoprotein abnormality, whereas others have a complex mechanism resulting in multimodal changes of the lipoprotein profile.

## Novelties in High LDL-Cholesterol Treatment

LDL-C lowering is the mainstay of drug treatment for high-risk patients. Statins, from older less powerful molecules such as pravastatin, have developed into more effective agents acting at lower doses (pitavastatin) and with potential LDL-C reductions of over 50% (rosuvastatin and atorvastatin) [18]. Besides the CV benefit shown in patients with coronary artery disease, the use of intensive lipid-lowering therapy with statins is also recommended after ischemic stroke of atherosclerotic origin. As recently reported in a parallel-group trial conducted in France and South Korea, among patients with evidence of cerebrovascular or coronary-artery atherosclerosis, those achieving a target LDL-C ≤ 70 mg/dL had a lower risk of major cardiovascular events compared to patients reaching an LDL-C range between 90 and 110 mg/dL [19].

In the attempt to reduce the ASCVD burden driven by LDL-C, as also corroborated by Mendelian randomization analyses [3], the use of inhibitors of the proprotein convertase subtilisin/kexin type 9 (PCSK9) has provided a very powerful additional therapy for hypercholesterolemia, allowing to set LDL-C targets ≤ 55 mg/dL, as in cases of secondary prevention in very high-risk patients or in familial hypercholesterolemia [8].

### PCSK9 Inhibition by Monoclonal Antibodies

The efficacy and safety of the two available monoclonal antibodies evolocumab and alirocumab have been extensively evaluated in the FOURIER and ODYSSEY OUTCOMES studies, respectively, as well as in the subsequent sub-analyses. Briefly, as reviewed by our group [20], in the FOURIER study enrolling patients with ASCVD, evolocumab was superior to placebo in reducing the major adverse cardiac events (MACEs) by 15%, an effect primarily driven by a decrement in MI (−27%), stroke (−21%) and coronary revascularization (−22%) [21]. Overall, although the benefit of evolocumab was similar across a broad range of ages and regardless of sex [22], patients who experienced a greater absolute risk reduction were those at higher risk for major vascular events e.g., those with greater atherosclerotic plaque burden[23], with peripheral artery disease [24] or with recent MI [25•, 26]. Finally, the benefit of adding PCSK9 to statins was evident also in patients with diabetes [27], metabolic syndrome [28], as well as in those with prior ischemic stroke [29].

In the case of alirocumab, the ODYSSEY OUTCOMES trial has shown the superiority of alirocumab vs placebo in reducing MACEs by 15% (a composite of cardiovascular death, non-fatal MI, fatal or non-fatal ischemic stroke, or unstable angina requiring hospitalization) in patients with an acute coronary syndrome. This benefit was evident irrespective of age [30] and independent of baseline eGFR, across a broad range above 30 mL/min/1.73 m^2^, with larger relative risk reductions in patients with eGFR> 60 mL/min/1.73 m^2^ [31]. Other major findings associated with alirocumab were the risk reduction of stroke (a benefit independent of the history of cerebrovascular disease [32]) and of total hospitalizations with a corresponding small rise in days alive and out of hospital [33].

In addition, when comparing the two trials, a stratification based on polygenic risk scores for coronary artery disease showed that individuals who derived a larger absolute and relative risk reduction were those with a high score [34, 35]. In a similar way, as reported in a recent meta-analysis of the two outcome trials, both monoclonal antibodies led to a 31% reduction in the risk of venous thromboembolism [36].

Finally, as elsewhere reviewed by our group [37], although there is no impact of PCSK9 monoclonal antibodies on high-sensitivity C-reactive protein (hsCRP), this biomarker identifies patients with a high CV risk, achieving better ASCVD prevention after treatment [38].

### PCSK9 Inhibition by Silencing RNA

**Fig. 1:**
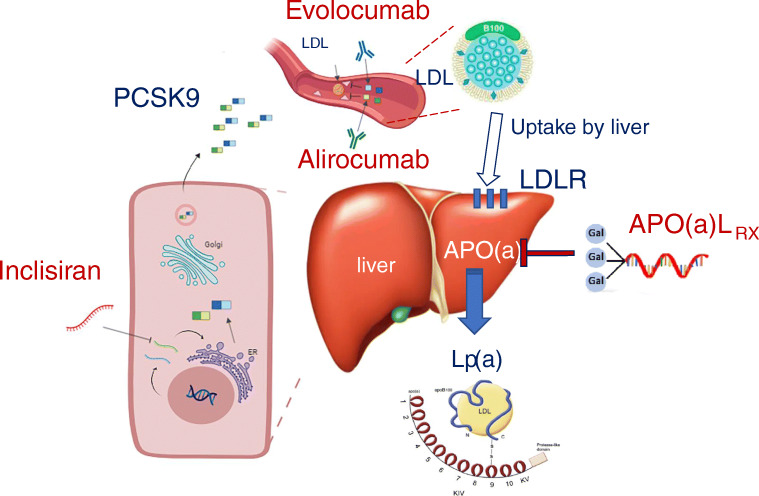
Molecular mechanisms of PCSK9 and Lp(a) inhibition. PCSK9, mainly derived from the liver, acts by degrading the LDLR expressed on hepatocytes. PCSK9 inhibition leads to higher LDLR expression, thus lowering plasma LDL cholesterol. Inclisiran by inhibiting the transcription of the PCSK9 mRNA reduces the total amount of circulating PCSK9. Conversely, monoclonal antibodies (evolocumab and alirocumab) block PCSK9 interaction with LDLR. Both therapies have shown to strongly reduce the LDL-C levels. Relative to Lp(a) lowering, IONIS-APO(a)Lrx is a ligand-conjugated ASO with a triantennary N-acetylgalactosamine (GalNAc) covalently attached, to allow rapid and specific uptake by hepatocytes. ASO, antisense oligonucleotide; LDLR, low-density lipoprotein receptor; Lp(a), lipoprotein(a); PCSK9, proprotein convertase subtilisin/kexin type 9. Adapted with permission of Elsevier [40].

A newer approach to inhibit PCSK9 has been provided by inclisiran, a siRNA currently under investigation. Inclisiran, specifically designed to target the 3′ UTR of the PCSK9 mRNA, is a long-acting siRNA whose 3′ end of the passenger strand is functionalized with triantennary GalNAc (review in [39]) (Fig. 1). Aside from the ORION 1 study whose positive results have been extensively reviewed elsewhere [41], the safety and efficacy of inclisiran have been evaluated by the still ongoing ORION program. ORION-10 (conducted in US) and -11 (conducted in Europe and South Africa) recruited patients with ASCVD or ASCVD risk-equivalent. The ORION-2, -5 and -9 trials have recruited instead familial hypercholesterolemia (FH) patients, whereas ORION-3 and -8 were single-arm, open label studies. Dose interval was 300 mg injected on day 1 and another dose on day 90, thereafter treatment being administered every 180 days (at day 270 and 450). Relative to this choice, it is worth mentioning that although inclisiran has a short plasma half-life (5–10 h), its long-lasting effect on LDL-C is possibly due to the entrapment of inclisiran in the endosomes which may serve as an intracellular depot of drug, slowly releasing it over time [42].

ORION-9 enrolled 482 heterozygous FH adult patients with mean baseline LDL-C of 153 mg/dL despite maximum tolerated lipid lowering drugs and followed for 18 months (510 days). A 1.5 mL sc injection of a 300 mg dose of inclisiran (corresponding to 284 mg of inclisiran free acid) led to an absolute change in LDL-C of −47.9% (95% CI, −53.5 to −42.3, p= 0.001) with a time-averaged percent change of −44.3% (between group difference). At day 510, the levels of circulating PCSK9 were decreased by 60.7% in the inclisiran group and raised by 17.7% in the placebo group. Treatment was associated with a higher percentage of local injection-related side effects vs placebo (17.0% vs 1.7%), the majority of events being rated as mild and none as serious. Low-titer antidrug antibodies were detected in 2.6% of the sample (25 samples from 18 patients), apparently not associated with changes in clinical endpoints [43].

In the US-based ORION-10 study, a 510-day treatment with inclisiran was superior to placebo in reducing LDL-C (−52.3%; 95%CI, 48.8 to 55.7). Among the serious adverse events, CV deaths were 7 (0.9%) in the inclisiran arm vs 5 (0.6%) in the placebo group, fatal/non-fatal MI were 21 (2.6%) with inclisiran and 18 (2.3%) with placebo. The prespecified exploratory CV end points were 58 (7.4%) in patients given inclisiran and 79 (10.2%) in those on placebo. Despite the same dramatic reduction in LDL-C (−49.9%; 95% CI, 46.6 to 53.1), in the non-US based ORION-11 trial, CV deaths were 9 (1.1%) with inclisiran and 10 (1.2%) with placebo, 10 fatal/non-fatal MI (1.2%) with inclisiran and 22 (2.7%) with placebo. The prespecified exploratory CV end points were 63 (7.8%) and 83 (10.3%), respectively. A meta-analysis on MACE in ORION-10 and -11 trials found that the benefit of LDL lowering achieved with inclisiran is in concordance with the results of 7 trials involving treatment with monoclonal PCSK9 inhibitors (the OSLER I–II trials, the ODYSSEY Long Term, the GLAGOV trial, the FOURIER trial, the SPIRE I–II and the ODYSSEY Outcomes trials) [44]. The still recruiting ORION-4 trial on coronary patients will clarify the CV benefit of this agent [45••] (Table 1).

**Table 1:** Ongoing outcome trials

	Outcome studies	Description
Inclisiran	ORION-4	A double-blind randomized placebo-controlled trial assessing the effects of inclisiran on clinical outcomes among people with atherosclerotic cardiovascular disease (NCT03705234)
Bempedoic acid	CLEAR Outcomes [46]	To determine if treatment with bempedoic acid (ETC-1002) versus placebo decreases the risk of cardiovascular events in patients who are statin intolerant (NCT02993406)
TQJ230 or APO(a)Lrx	Lp(a)HORIZON	Assessing the impact of lipoprotein (a) lowering with TQJ230 on major cardiovascular events in patients with CVD (NCT04023552)
Pemafibrate	PROMINENT [47]	The primary objective of the study is to determine whether pemafibrate administered twice daily will delay the time to first occurrence of any component of the clinical composite endpoint of non-fatal myocardial infarction, non-fatal ischemic stroke, hospitalization for unstable angina requiring unplanned coronary revascularization or cardiovascular death (NCT03071692)

Finally, ORION-7 (conducted in New Zealand) was a phase 1, open-label study evaluating the effect of renal impairment on pharmacokinetics (PK), pharmacodynamics (PD), safety, and tolerability of a single s.c. dose of inclisiran sodium 300 mg. Since renal clearance is the main route of elimination of inclisiran, with approximately one-third of the total administered does detected in urine within 24 h, patients were allocated to four groups based on renal function: normal (creatinine clearance – CrCl ≥90 mL/min), mild (CrCl 60–89 mL/min), moderate (CrCl 30–50 mL/min), and severe (CrCl 15–29 mL/min) renal impairment. The renal clearance of inclisiran was reduced proportionally to the degree of renal impairment. Relative to PD effects or safety profile, there were no differences among individuals with normal renal function and patients with mild, moderate or severe renal impairment. Moreover, at day 60, the plasma levels of PCSK9 were reduced by 68.1%±12.4% in the group with normal renal function, by 74.2%±12.3%, 79.8%±4.9%, and by 67.9%±16.4%, respectively, in those with mild, moderate, and severe renal impairment [48].

### Bempedoic Acid

This compound is a linear chain molecule acting as a selective antagonist of ATP citrate lyase (ACLY), an important step in both fatty acid and cholesterol biosynthesis. Inhibition of ACLY leads, in fact, to a reduction of acetyl-CoA, the direct precursor of HMG-CoA, thus leading to an increased activity of LDL-receptors with reduction of plasma cholesterol [49]. In addition, bempedoic acid activates AMPK in different cell types potentially leading to an improvement in insulin resistance [50], with the potential of not increasing the diabetes risk. Pooled data from four phase 3 clinical trials reported that a new-onset diabetes/hyperglycemia occurred less frequently with bempedoic acid vs placebo [51].

Bempedoic acid is a prodrug, activated by acyl-CoA synthetase-1 only in the liver, thus excluding the potential of exerting any action on skeletal muscle [49]. Indeed, looking at the clinical trials, there is essentially no occurrence of significant muscular side effects [52]. As recently reported, the analyses of phase 3 trials (CLEAR Tranquility, CLEAR Harmony, CLEAR Wisdom, and CLEAR Serenity) showed that bempedoic acid reduced LDL-C roughly by 18% when given on-top of statins and by 24% when given as a monotherapy [53]. The drug is also available in a fixed dose combination (180 mg) with ezetimibe (10 mg) leading to potential LDL-C reduction of 41% [54]. Similar to statins, treatment with bempedoic acid markedly reduces hsCRP levels [38]. Relative to safety, a modest increase in blood urea nitrogen, creatinine, and uric acid and a decrement in hemoglobin were found in treated patients. A raised incidence of gout was found with bempedoic acid (1.6/100 person-years) vs 0.5/100 person-years for those allocated to placebo [51]. A very recent meta-analysis of CV outcomes from 4 studies on a total of 3,483 patients indicated 17%, non-statistically significant reduction of five-point MACE with, however, a surprising 50% lowering of non-coronary revascularizations [55]. On this matter, although a Mendelian randomization analysis predicted a reduction in CVD risk per unit decrease in the LDL-C levels in carriers of loss-of-function mutation in *ACLY* similar to that obtained in carriers of loss-of-function for *HMGCR* [56], as yet no clinical reports of adequate duration on CV event reduction have been published. A large secondary prevention study in 12,000 statin intolerant patients is ongoing and should be reported in the first quarter of 2022 [46] (Table 1).

### Angiopoietin-like Protein 3

A somewhat unexpected development in the field of cholesterol lowering medications has been that of antagonists of angiopoietin-like protein 3 (ANGPTL3) [57]. ANGPTL3 inhibits hydrolysis of TGs by lipoprotein lipase (LPL) and loss of function mutations in the ANGPTL3 gene are associated with protection against ASCVD [40, 58]. Treatment with evinacumab, a fully human monoclonal antibody directed to ANGPTL3, led to reduced plasma TG and surprisingly LDL-C levels in humans [59]. Of particular interest has been the observation that inhibition of ANGPTL3 significantly reduced LDL-C in homozygous FH (HoFH) patients [60]. In the earlier series, in nine HoFH patients, including two LDL receptor gene *(LDLR)* null/null homozygotes, the mean LDL-C reduction after 4 weeks on evinacumab (250 mg sc at baseline and 15 mg/kg iv at week 2) was 49±23%. These patients were already on optimal treatment with statins, ezetimibe, lomitapide, and PCSK9 monoclonal antibodies. The two null/null homozygotes showed a modest response. More recently, a larger study (ELIPSE trial) on 65 homozygotes showed that iv infusion of evinacumab (15 mg/kg), every 4 weeks, followed for 24 weeks reduced LDL-C by 47.1% compared with a 1.9% increase in the placebo group (between group mean difference of −49%) [61]. Responses were robust even in patients with *LDLR* null/null variants (−43.4% vs. +16.2% in placebo). No significant differences in adverse events between those receiving evinacumab or placebo were reported.

The superiority of evinacumab administered either sc or iv compared to placebo was also seen in patients with refractory hypercholesterolemia. Among 272 patients with LDL-C levels of 150±80.2 mg/dL despite use of other lipid-lowering medications including monoclonal PCSK9 inhibitors, evinacumab significantly reduced LDL-C by more than 50% at the maximum dose (450 mg/weekly sc regimen and 15mg/Kg every 4 weeks iv regimen) [62]. The pharmacokinetic profile of both regimens seems not to differ between ethnicities at least in the Caucasian and in the Japanese [63].

## Lp(a)—Novelties in the Era of RNA-based Therapies

Despite the association between Lp(a) elevations and coronary artery disease risk has emerged from both epidemiological and genetic studies [64–66], a pharmacological approach able to lower Lp(a) levels to the extent required to potentially achieve a CV benefit in patients with progressive ASCVD and high plasma Lp(a) is still missing (reviewed in [67])**.** Available lipid-lowering therapies have little value for Lp(a) reduction. Aside from statins that could raise Lp(a) blood concentrations [68, 69], PCSK9 inhibitors reduce Lp(a) levels by 25–30% [70] and there is evidence that reduction of Lp(a) elicited by these drugs might contribute to an improved prognosis in coronary disease patients [71]. Finally, for patients with progressive ASCVD and high plasma Lp(a), a possible approach sometimes used is lipoprotein apheresis [72]*.* Although with the limitation of not being a randomized trial, the prospective study by Roeseler et al. [73] reported a significant CV benefit with different apheresis techniques in patients with hyperlipoproteinemia(a), an observation reported also in the German Lipoprotein Apheresis Registry [74].

In view of the limited activity of generally available lipid-lowering drugs and the ongoing progress in antisense-DNA based therapies, the most recent phase 2b trial with an apo(a) antisense oligonucleotide (ASO) tested an APO(a)Lrx conjugated with GalNac in patients with pre-existing CVD and baseline Lp(a) > 60 mg/dL. The active compound reduced dramatically, in a dose-dependent manner, the levels of Lp(a): mean percentage decreases were 35% at 20 mg Q4W, 56% at 40 mg Q4W, 58% at 20 mg Q2W, 72% at 60 mg Q4W, and 80% at 20 mg QW. These changes allowed 97.7% of patients allocated to 20 mg QW to reach Lp(a) levels ≤ 50 mg/dL. APO(a)Lrx was well tolerated and did not lead to changes in platelet counts, liver and renal functions, with no signs of influenza-like symptoms. The most common associated adverse reaction was erythema (26%) and only one patient discontinued treatment because of an injection site reaction [75].

Very recently, the activity of a new GalNAc-conjugated small interfering RNA targeting mRNA transcribed from the *LPA* gene (Amg890) has been reported in Abstract form. In this phase 1 trial (NCT03626662), in 64 patients with elevated Lp(a), a single-dose treatment with AMG 890 was well-tolerated and significantly reduced Lp(a) with observed maximal percent reductions of > 90% [76]. A phase 2 study is ongoing (NCT04270760) with an estimated enrollment of 240 patients with ASCVD and Lp(a) >150 mg/dL. Completion is expected by April 2023.

## Triglyceride Reduction: New Options and Effectiveness

Lowering of TGs is today still best accomplished by peroxisomal proliferator-associated receptor (α, β, and γ) activation. Fibrates, the most effective activators of the PPARα system, generally have a mixed PPARα, β activity [77]. PPARs belong to the nuclear hormone receptor superfamily and, by binding to PPAR-response regulatory elements (PPRE) heterodimerize with the retinoid X receptor (RXR) thus modulating genes involved in adipogenesis, lipid metabolism, inflammation, and control/maintenance of metabolic homeostasis [78, 79]. Among fibrates, fenofibrate is mainly active on PPARα whereas bezafibrate acts on PPARα-β/δ, glitazones acting instead on PPARγ . By this mechanism, fibrates down-regulate apolipoprotein-CIII (apo-CIII) while stimulating the lipoprotein lipase gene expression thus affecting TG metabolism [80].

Among PPARs, PPARα (also called NR1C1) is activated mainly under energy deprivation, in particular during fasting [81], PPARα -mediated fatty acid catabolism is crucial for the synthesis of metabolites to be used as energy sources to other tissues such as ketone bodies in the brain [82]. In addition to activating a number of genes in the FA β-oxidation pathway, e.g., carnitine palmitoyl transferase 1A and 2, PPARα also exerts an anti-inflammatory activity in mouse models, although contrasting data have been reported [83].

### Pemafibrate

Currently, there is growing interest in the selective activation of PPARα. Newly available PPARα agonists may provide an advancement, since older fibrates have a relatively weak activity and display limited efficacy due to elevation of transaminase, homocysteine, and creatinine with consequent, although rare, myopathy. The increased selectivity of PPARα is best exemplified by pemafibrate (previously known as K877), >2,000-fold more selective for PPARα vs either PPARγ or δ [84, 85]. Clinical evaluation confirmed the extremely low active doses (0.1–0.2 mg bid) compared to fenofibrate (106.6 mg qd). A comparative trial over 24 weeks showed TG reductions of 46% for pemafibrate vs −39.7% for fenofibrate [86]. This finding was confirmed in a number of further trials, also indicating that adverse events were less frequent vs fenofibrate 200 mg/day [87]. The use of pemafibrate was also proposed for the handling of residual dyslipidemia for patients with TG>300 mg/dL on statins. Regardless of statin background combination therapy, pemafibrate 0.2–0.4 mg/day led to TG reductions of about 50% from baseline [88]. These very positive findings have led to the planning of the PROMINENT (Pemafibrate to Reduce Cardiovascular Outcomes by Reducing Triglycerides in Patients with Diabetes) trial involving high ASCVD risk patients with in use of T2DM on statin therapy and who persist with atherogenic dyslipidemia. The primary end-point is the time of occurrence of the first non-fatal MI, ischemic stroke, unstable angina or coronary revascularization, and CV deaths [47]. Two studies in Europe and the USA are currently ongoing in patients with severe hypertriglyceridemia (Table 2).

**Table 2:** Ongoing or recently completed studies involving inhibition of agents reducing triglyceride-rich lipoproteins

	Trial	Description
Pemafibrate	NCT03011450 (phase 3, in Europe) and NCT03001817 (phase 3, in US)	Study to evaluate the efficacy and safety of K-877 in adult patients with fasting high triglyceride levels and mild or moderate renal impairment
Evinacumab (mAb against ANGPTL3)	NCT04233918 (phase 3)	Evaluating the efficacy and safety of evinacumab in pediatric patients with homozygous familial hypercholesterolemia
	NCT03452228 (phase 2)	Safety and efficacy following repeat-dose of evinacumab (Anti-ANGPTL3) in patients with severe hypertriglyceridemia (sHTG) at risk for acute pancreatitis
	NCT03409744 (phase 3)	Evaluate the long-term safety and efficacy of evinacumab in patients with homozygous familial hypercholesterolemia
Vupanorsen (ANGPTL3-L_Rx_)	NCT04516291 (phase 2)	A dose-ranging study with vupanorsen (TRANSLATE-TIMI 70)
	NCT04459767 (phase 1)	Investigation of safety, tolerability, pharmacokinetics and pharmacodynamics of single doses of vupanorsen in Japanese healthy adult participants with elevated triglycerides
ARO-ANG3 (silencing RNA)	NCT03747224 (phase 1)	Study of ARO-ANG3 in healthy volunteers and in dyslipidemic patients
AKCEA-APOCIII-L_Rx_	NCT04568434 (phase 3)	A study of administered to patients with familial chylomicronemia syndrome (FCS) (BALANCE)
	NCT03385239 (phase 2)	Study of ISIS 678354 (AKCEA-APOCIII-LRx) in patients with hypertriglyceridemia and established cardiovascular disease (CVD)
ARO-APOC3 (silencing RNA)	NCT03783377 (phase 1)	Study of ARO-APOC3 in healthy volunteers, hypertriglyceridemic patients and patients with familial chylomicronemia syndrome (FCS)

### Omega-3

The latest development in the treatment in hypertriglyceridemias has been the unexpected CV effectiveness of FA of the n-3 series (i.e., with multiple double bonds, the first being in the n-3 position from the terminal methyl group). These omega-3s act as “fraudulent fatty acids” [89], i.e., they, somewhat similar to drugs with the FA-like structure, particularly fibrates, do not follow the liver metabolic handling by the classical fatty acetyl CoA oxidative mechanism with carnitine-mediated transport to mitochondria [90]. They exert, instead, a moderate stimulation of the PPARα -mediated pathway, although peroxisomal proliferation is less extensive than in the case of fibrates [91]. Clinical trials in patients given elevated daily doses of omega-3 in the form of TG or more recently of ethyl esters of eicosapentaenoic (EPA) or docosaexaenoic (DHA) acid, as well as with novel formulations of separated fatty acids, repeatedly confirmed an effective activity in TG reduction, particularly in patients with diabetes [92]. A general review on the mechanisms of omega-3 involves targeting of characteristics of the metabolic syndrome, i.e., raised adipocyte differentiation, reduced lipolysis and lipogenesis and, more recently, reduced inflammatory changes in the adipose tissue, characteristic of obesity [93]. Recent evidence has, however, provided exciting observations from a clinical trial on high dose EPA. The REDUCE-IT trial involved 8,179 participants with high CV risk (71% with established CV disease and 58% with T2DM). These had essentially normal LDL-C upon optimal statin treatment (75 mg/dL) whereas mean TG levels were moderately elevated (median value 216 mg /dL). Patients receiving 4 g of icosapent ethyl per day (2 g bid with meals) vs placebo for a median follow-up of 4.9 years had an absolute between group difference in primary CV endpoints of 4.8% vs placebo with a number needed to treat of 21 [94]. Interestingly, a subsequent evaluation of the trial reported a significant decrement in the first and total CV deaths [95]: total primary endpoint events (CV death, nonfatal myocardial infarction, nonfatal stroke, coronary revascularization, or hospitalization for unstable angina) were decreased by 30% and total key secondary endpoint events (cardiovascular death, nonfatal myocardial infarction, or nonfatal stroke) by 28%. Findings of the REDUCE-IT trial indicate that TG reduction (e.g., −18.3% fall from baseline to 1 year) may be an important target of therapy, although possibly not all the benefits reported in the trial are explained by TG lowering. This tentative conclusion is supported by the recently reported EVAPORATE (Effect of Vascepa on Improving Coronary Atherosclerosis in People With High Triglycerides Taking Statin Therapy) trial in 80 coronary artery disease patients. They were allocated into two groups given a similar dose of EPA as in the REDUCE-IT trial or a placebo and followed for 18 months. Coronary computed tomographic scans showed that icosapent ethyl was superior to placebo in reducing plaque volume (primary endpoint) by 17%, fibrofatty and fibrous plaques by −34% and by −20%, respectively, whereas dense calcium did not change between groups [96].

Differently from the icosapent ethyl formulation, daily administration of 4 g of the carboxylic acid formulation of EPA and DHA, in patients with hypertriglyceridemia (> 240 mg/dL) and or diabetes (70%) resulted in neutral effects on CV prevention compared to corn oil (placebo). Despite a −20% reduction in TG and hsCRP, the HR for MACE (a composite of CV death, nonfatal MI, nonfatal stroke, coronary revascularization, or unstable angina requiring hospitalization) was 0.99 (95%CI 0.90–1.09). The STRENGTH (The Long-Term Outcomes Study to Assess Statin Residual Risk with Epanova in High Cardiovascular Risk Patients with Hypertriglyceridemia), recruiting 13,078 patients, was halted prematurely when it became apparent that the probability of clinical benefit was likely to be low. An increased rate of new-onset atrial fibrillation (HR 1.69, 95%CI 1.29–2.21) and of gastrointestinal adverse events [97] was observed. Compared to the REDUCE-IT study, the achieved EPA levels in plasma and red blood cells were lower but it is uncertain whether these differences would be sufficient to explain the impact on MACE. The question as to whether possible deleterious effects of DHA in STRENGTH or the use of mineral oil in REDUCE-IT, with the potential of the latter to raise LDL-C and hsCRP, could be responsible for the different findings remains to be determined [98] although data from comparative trials suggest no significant impact of mineral oil on CV outcomes [99].

### ANGPTL3 and Apo C-III Inhibition

In addition to evinacumab, antisense oligonucleotides (ASOs) targeting ANGPTL3 messenger RNA are under clinical evaluation. ANGPTL3-L_Rx_, a second-generation ASO drug targeting ANGPTL3 mRNA, has a covalent linkage with the GalNAc cluster, conferring high affinity for the hepatocyte-specific asialoglycoprotein receptor (reviewed in [40]).

A phase 1 trial aimed at testing the safety, pharmacokinetics, and pharmacodynamics of single ascending doses and multiple ascending doses of ANGPTL3-L_RX_ (vupanorsen) in healthy volunteers showed that this last was the regimen to be used. Once a week injection for 6 weeks of active compound reduced, at day 43, the circulating levels of ANGPTL3 from baseline by 46.6% (10 mg), 72.5% (20 mg), 81.3% (40 mg), and 84.5% (60 mg). Compared to placebo (0.9% sterile saline s.c.), ANGPTL3-L_RX_ lowered TG (from −33.2 to −63.1%), LDL-C (from −1.3 to −32.9%), VLDL-C (from −27.9 to −60%), non-HDL-C (from −10 to −36.6%), apoB (from −3.4 to −25.7%), and apolipoprotein (apo)C-III (from −18.9 to −58.8%). No clinical signs of prothrombotic effects, bleeding episodes, and significant decreases in platelet counts and liver or renal function damages were found ([100]).

In a recent phase 2 trial, enrolling 105 patients with median TG levels of 252 mg/dL and T2DM, vupanorsen reduced ANGPTL3 by 41% (40 mg Q4W), 59% (80 mg Q4W), and 56% (20 mg QW) leading to a dramatic reduction in TG levels, −36% (40 mg Q4W), −53% (80 mg Q4W), and −47% (20 mg QW). Six months of treatment allowed most of the patients to reach TG levels < 150 mg/dL: 35% (40 mg Q4W), 58% (80 mg Q4W), and 39% (20 mg QW). At the dose of 80 mg Q4W, ANGPTL3 was reduced by 62%, apoC-III by 58%, remnant cholesterol (and VLDL-C) by 38%, non-HDL-C by 18%, TC by 19%, apoB by 9%, and HDL-C by 24%. Changes in LDL-C (−12%) were found only in patients given the dose of 20 mg QW. The most frequent adverse effects were injection-site pruritus and erythema, whereas no one experienced a confirmed platelet count <100,000/mm^3^ [101••].

Besides ANGPTL3, another recently approached target for the treatment of severe hypertriglyceridemia is provided by inhibitors of apo C-III, a multifaceted protein in cardiometabolic disease [102]. Apo C-III is an inhibitor of LPL and has been recently identified as a risk factor for CV disease [103, 104]. Apo C-III is a small molecule with a molecular weight of 8.8 kDa and 79 amino-acids, rapidly exchanged postprandially between lipoproteins [105]. Indeed, all lipoproteins may contain ApoC-III, although this apoprotein is mainly represented in chylomicrons, VLDL, their remnants, and HDL. The clinical observation of apo C-III mutations with significant loss-of-function and associated with low TGs and elevated HDL has been reported in a number of studies [106]. The lead investigators described a potential therapeutic approach to hypertriglyceridemia based on an inhibited activity of apo C-III (reviewed in [107]).

At present, the only reported effective strategy is by administration of an antisense oligonucleotide, although monoclonal antibodies STT505 and STT5058 seemed to lower apo C-III levels and promote clearance of TG-rich lipoproteins in mice [108].

The antisense inhibition was first evaluated by Gaudet et al. in patients with triglyceridemia between 350 and 2,000 mg/dL on stable fibrate therapy. Volanesorsen at doses from 100 to 300 mg once weekly for 13 weeks led to dose-dependent reductions of apoC-III (from −40 to −79.6%) concomitant with dramatic TG reductions (from 31.3 to 70.9%) [109]. A larger study was conducted on 66 patients with familial chylomicronemia syndrome (FCS) treated for 52 weeks. Volanesorsen dramatically reduced apoC-III levels by 84% and mean TG by 77%, allowing after 3 months 77% of the patients to achieve TG levels ≤ 750 mg/dL. However, 14 patients given volanesorsen did not terminate the trial due to platelet count reductions [110]. In spite of this drawback, the European Medical Agency (EMA) considered positively the benefit: risk ratio thus authorizing volanesorsen for the treatment of FCS. A recent meta-analysis of the available phase 2 and phase 3 clinical studies reported that volenesorsen significantly reduced VLDL-C (−73%), TG (−68%), ApoCIII (−74%), and raised HDL (+40%) and LDL-C (+47%, p= 0.057). Specifically, looking at LDL-C, it is worth mentioning that although in the APPROACH study the rise in LDL-C was +136%, the basal LDL-C levels were very low 28±19 mg/dL to become 61±39 mg/dL, still remaining in the normal range [110]. Overall, the increment in LDL-C may be consequent to an enhanced conversion of VLDL to LDL or due to changes in the secretion and catabolism of the LDL particles, although changes in CETP activity cannot be excluded [111].

Injection of volanesorsen was associated with a significantly higher risk of injection site reactions (OR= 32.89, 95%CI= 7.97–135.74) when compared to placebo [112].

Recently, a new GalNac-conjugated APOCIII-L_Rx_ was tested in a dose-escalation Phase 1/2a study in healthy volunteers. A large improvement in the atherogenic lipid profile was detected both following a single-dose- or a multi-dose-regimen. This last choice led to median reductions of apoC-III by 66% (15 mg QW4), 84% (30 mg QW4), and by 89% (60 mg QW4) and of TG by 59%, 73%, and 66%, respectively. No flu-like reactions, platelet count reductions, liver, or renal safety signals were reported [113, 114].

## Conclusions

**Fig. 2:**
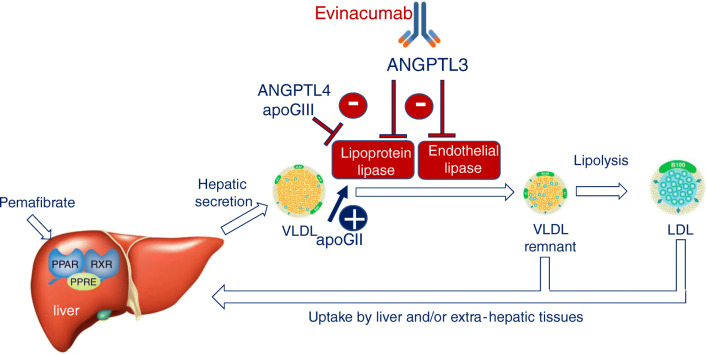
Molecular mechanism of ANGPTL3 inhibition. ANGPTL3 inhibits both the lipoprotein and endothelial lipases and thus the conversion of VLDL into LDL. A similar effect is determined by ANGPTL4 and apoC-III, while apoC-II is a coactivator of lipoprotein lipase. The monoclonal antibody evinacumab binds and inhibits the ANGPTL3 generating VLDL remnant particles that are efficiently removed from the circulation. Thus, ANGPTL3 inhibition may lower LDL-C by limiting LDL particle production and endothelial lipase could be a key mediator of this novel pathway. ANGPTL, angiopoietin-like; apo, apolipoprotein; LDL, low-density lipoprotein; VLDL, very-low density lipoprotein. Adapted with permission of Elsevier [40].

The considerable progress in developing newer drugs and strategies for the management of hyperlipidemias will probably lead to considerable improvement in the clinical practice in the near future. New agents are now taking optimal care of FH, even of the homozygous phenotype both in adults and pediatric individuals [115, 116]. In this context, the enigmatic activity of evinacumab, an antagonist of ANGPTL3, to ameliorate LDL-C in HoFH patients may become of interest if statins, cholesterol-absorption inhibitors, or PCSK9 inhibitors would not be able to lower LDL-C to a sufficient degree or have unacceptable side effects. Specifically, the effect of evinacumab on extremely elevated LDL-C levels has been partly clarified by two different groups, both indicating that endothelial lipase (EL) may mediate the LDLR independent effect of ANGPTL3 inhibition. Adam et al. [117] reported that in the absence of EL, no significant LDL-C reduction is observed. In line with this evidence, Wu et al. [118] noted that in EL knock-out mice, compared to wildtype ones, ANGPTL3 inhibition reduced LDL-C only in the latter. Although a clear mechanism linking ANGPTL3 inhibition and LDL-C reduction remains unsolved as yet, the most reliable hypotheses report an EL-mediated clearance of LDL precursors (VLDL and IDL) or an EL-accelerated LDL catabolism (Fig. 2).

The importance of reducing LDL-C has been further underscored by a recent prospective study on 4958 asymptomatic adults evaluating the relationship among the incidence of ASCVD event risk, the cumulative exposure to LDL-C, and the time course of LDL-C accumulation [119]. However, moving to real-life, there is a gap between clinical guidelines and clinical practice, an evidence underscored by the recent DA VINCI study, an 18 country, cross-sectional, observational study of patients prescribed lipid-lowering therapies for primary and secondary care across Europe [120••].

The recent 2019 European guidelines for the management of hypercholesterolemia have added PCSK9 inhibitors to the therapeutic armamentarium to achieve the LDL-C goals. Besides monoclonal antibodies, a new biosynthetic drug is approaching, inclisiran. However, despite the powerful effect on LDL-C lowering, a recent cost-effectiveness analysis from the Australian healthcare perspective showed that the cost of inclisiran would have to be 60% lower than that of evolocumab [121]. This observation is of high interest considering that the 2019 ESC/EAS guidelines rendered half of all post-acute coronary syndrome patients potentially eligible for PCSK9 inhibitors [122]. Thus, in order to achieve the LDL-C target levels in high risk ASCVD patients, adding new drugs like bempedoic acid, more cost-effective but less potent than PCSK9 inhibitors, may be envisioned as second- or third-line agents, similar to ezetimibe and bile acid sequestrants (reviewed in [84]).

Notwithstanding, major challenges are forthcoming in the case of Lp(a), a still not fully understood CV risk marker. Two generations of ASOs have been presented, apparently allowing to reduce Lp(a) by up to 90%. In this special case, long term-controlled risk evaluations will also allow to clarify the independent risk role of elevated Lp(a).

Since the pathophysiology of atherosclerosis is a continuum from early to advanced vascular disease with lipoproteins playing a pillar role, it is worth mentioning that in ASCVD patients carrying the traits of metabolic syndrome, a considerable residual risk remains. Considering that the most recent approaches to manage severe forms of hypertriglyceridemia by means of the antagonism of apo C-III or of ANGPLT3 are far from to be clinically available, the use of icosapent ethyl or pemafibrate [123] becomes of high interest. In individuals with high levels of TG, icosapent ethyl was superior to placebo to reduce not only the first CVD event but also the subsequent ones [124]. This evidence is of extreme importance considering that CVD risk is eight-fold higher among those with ASCVD than those without it [125]. However, considering that the global market for omega-3 fatty acids has reached $4.1 billion and is expected to double by 2025, the neutral effects reported in the STRENGTH and VITAL (Vitamin D and Omega-3 Trial) studies should be a reminder that the widespread use of over-the-counter mixed omega-3 products lacks evidence for clinical utility [126].
